# Cytomegalovirus seroprevalence, infection, and disease in Chinese thoracic organ transplant recipients: a retrospective cohort study

**DOI:** 10.1186/s12879-022-07853-x

**Published:** 2022-11-22

**Authors:** Chunrong Ju, Xiaohua Wang, Xin Xu, Shaobo Xie, Qingdong Cao, Wanli Lin, Jianheng Zhang, Yu Xu, Qiaoyan Lian, Danxia Huang, Rongchang Chen, Jianxing He

**Affiliations:** 1 Guangzhou Institute of Respiratory Health, First Affiliated Hospital of Guangzhou Medical University, Yanjiang Road, Guangzhou, China; 2 Department of Thoracic Surgery and Lung Transplantation, Fifth Affiliated Hospital of Sun Yat-sen University, Zhuhai, China; 3 Department of Thoracic Surgery, The People’s Hospital of Gaozhou, Gaozhou, China; 4 Shenzhen People’s Hospital Institute of Respiratory Diseases, Southern University of Science and Technology, University of Jinan Second Clinical Medical College, East of Shennan Road, Luohu District, Shenzhen, China

**Keywords:** Cytomegalovirus, Thoracic organ transplant, Valganciclovir, Pneumonia, Seroprevalence

## Abstract

**Background:**

Cytomegalovirus (CMV) infection is a leading cause of morbidity and mortality after transplantation. This study aimed to investigate CMV seroprevalence, infection, and disease in Chinese thoracic organ transplant recipients.

**Methods:**

The clinical data of the patients who underwent lung and/or heart transplantation between January 2015 and October 2020 were retrospectively collected from four transplantation centers in China.

**Results:**

A total of 308 patients were analyzed. The CMV serostatus was donor positive (D^+^) recipient negative (R^−^) in 19 (6.17%) patients, D^+^/R^+^ in 233 (75.65%), D^−^/R^+^ in 36 (11.69%), and D^−^/R^−^ in 20 (6.50%). CMV DNAemia was detected in 52.3% of the patients and tissue-invasive CMV disease was diagnosed in 16.2% of the patients. Only 31.8% of the patients adhered to the postdischarge valganciclovir therapy. The D^+^/R^−^ serostatus (odds ratio [OR]: 18.32; 95% confidence interval [CI]:1.80-188.68), no valganciclovir prophylaxis (OR: 2.64; 95% CI: 1.05–6.64), and higher doses of rabbit anti-human thymocyte globulin (> 2 mg/kg) (OR: 4.25; 95% CI: 1.92–9.42) were risk factors of CMV disease.

**Conclusion:**

CMV seroprevalence was high in Chinese thoracic organ transplant donors and recipients. The low adherence rate to the postdischarge CMV prophylaxis therapy in Chinese patients is still an unresolved issue.

## Introduction

Organ transplantation is often the last treatment option for end-stage heart and lung disease. The number of thoracic organ transplantation is increasing rapidly in recent years, according to the International Society for Heart and Lung Transplantation registry [1]. However, posttransplant cytomegalovirus (CMV) infection remains a significant contributor to overall morbidity and mortality in thoracic organ transplant recipients [2]. Moreover, CMV infection has indirect impacts on the allografts, leading to adverse outcomes such as chronic allograft dysfunction and cardiac allograft vasculopathy [3]. Compared to other solid organ transplant recipients, thoracic organ transplant recipients are at greater risk of CMV infection because the lung is the principal reservoir of latent CMV and higher doses of posttransplant immunosuppressants.

Donor-derived CMV is a common cause of recipient infection after solid organ transplantation [4, 5]. The risk of CMV infection vary according to the CMV serostatus in donors and recipients. CMV-seronegative recipients (R^−^) of CMV-seropositive donors (D^+^) have higher risk of posttransplant CMV infection. CMV prophylaxis with antiviral agents such as valganciclovir or ganciclovir for 6 to 12 months is now a standard of care for thoracic organ transplant recipients [6].

China has witnessed a rapid development of thoracic organ transplantation, with 1053 lung transplants completed through 2015 to 2018 and an average annual growth rate of 35% [7]. However, there is a paucity of data of CMV infection and its prophylaxis in Chinese thoracic organ transplant recipients. The present study aimed to investigate CMV seroprevalence and identify the risk factors of CMV disease in this population.

## Methods

### Recipients

This study was a retrospective cohort study. We collected data on all the thoracic organ transplant recipients receiving transplantation between January 2015 and October 2020 at four centers in China. The inclusion criteria were: (1) age ≥ 18 years; (2) single or double lung transplantation, heart transplantation, or heart-lung transplantation. Patients with missing data due to incomplete medical history or lost to follow-up were excluded.

### Donors

Voluntary citizen-based deceased organ donation system was adopted in January 2015 in China [7]. Since then, the civilian organ donation has been the sole source for organ transplantation in China. Written informed consent for organ procurement was obtained from the living donors or from the family members of the brain death donors and the cardiac death donors. On the day before organ procurement, the peripheral blood was collected and the plasma CMV viral load (IU/ml) was measured by using a commercial quantitative nucleic acid testing kit (ABI 7500 real-time fluorescence quantitative PCR, Dietu Biotechnology Co., Ltd, Shanghai, China). Donors with a CMV viral load > 500 IU/ml were excluded from organ procurement [7, 8].

### Immunosuppressive scheme

All four transplant centers adopted a standardized immunosuppressive scheme including an induction therapy and a triple immunosuppression maintenance therapy consisting of a calcineurin inhibitor (cyclosporin A or tacrolimus), mycophenolate sodium or mycophenolate mofetil, and oral prednisolone [6]. Tacrolimus was dosed to get an ideal target level based on the therapeutic drug monitoring. Methylprednisolone 500 mg at induction and oral or injected steroids titrated to be maintained at 0.25 mg/kg thereafter. Induction therapy with basiliximab or rabbit anti-human thymocyte globulin (r-ATG) was used on a case-by-case basis. Basiliximab 20 mg was administered on day 0 and day 4. The dose of r-ATG was prescribed individually for treatment of rejection after transplantation.

### Data collection

The following data were collected: age of the donors and recipients at transplantation; date of transplantation; weight and height at transplant; CMV IgG serostatus and DNA loads of the recipients and donors; human leukocyte antigen mismatches; induction therapy (interleukin-2 receptor antibody or T cell depleting antibody); immunosuppressives prescribed at 0, 3, 6 months and at each year posttransplant; patient survival; prophylaxis and treatment for CMV DNAemia and CMV disease.

### CMV monitoring and prophylaxis

Plasma CMV viral loads in the recipients were monitored weekly as part of the routine viral surveillance using the PCR method, from induction until 60 days post-transplantation, and thereafter monthly until 6 months, and then once every 1.5–2 months until 1 year after transplantation. Genotypic assay for *UL97* and *UL54* mutations conferring ganciclovir resistance were performed by using the real-time polymerase chain reaction assay [9, 10]. Bronchoscopy was performed if the patients showed CMV disease symptoms and the physician deemed it necessary. CMV viral loads in the bronchoalveolar lavage fluid (BALF) were routinely measured using the PCR method with a detection limit of 500 IU/ml if the sample was available. All patients were followed up from the day of transplantation until death or January 2021.

All recipients received CMV prophylaxis with intravenous ganciclovir 5 mg/kg twice daily during postoperative day 1–14, followed by intravenous ganciclovir 5mg/kg once daily until discharge. Upon discharge, oral valganciclovir 450 mg once or twice daily was prescribed for 6 months. The dosage was adjusted according to creatinine clearance rate and body weight.

### Diagnosis of CMV infection

CMV infections were divided into asymptomatic CMV DNAemia and tissue-invasive CMV disease [6]. CMV DNAemia were defined as a plasma CMV DNA level > 500 IU/ml, which also indicated the start of antiviral therapy. The first episode of CMV DNAemia detected in each recipient was analyzed in this study. Definitive diagnosis of tissue-invasive CMV disease was made by immunohistochemistry in the biopsies, with the exception of CMV retinitis.

CMV pneumonia was classified into proven or probable disease as defined by Ljungman et al. [10]. Proven CMV pneumonia required histopathological evidence (i.e., viral inclusions and immunohistochemical staining) in the lung tissues. Probable CMV pneumonia was diagnosed based on clinical symptoms such as fever, cough, dyspnea, hypoxia, and muscle soreness, and CMV DNAemia and compatible pulmonary computed tomography (CT) findings, excluding other potential causes for these findings [10].

CMV retinitis was diagnosed according to the criteria proposed by the Standardization of Uveitis Nomenclature Working Group [11]. CMV myocarditis was diagnosed in patients who met the following criteria: 1) arrhythmia and elevated levels of myocardial enzymes that have no other explanations; 2) evidence of immune compromise; 3) detection of CMV DNAemia.

### Statistical analysis

Statistical analysis was performed by using SPSS 19.0 (IBM Corp., Armonk, NY, USA). The data were plotted by using GraphPad Prism 5 (GraphPad Software, Inc., San Diego, CA, USA). Normally distributed continuous data were expressed as mean ± standard deviation and analyzed by using the independent samples t-test. Differences between patients with and without CMV disease were analyzed by using the Chi-square test or the Fisher’s exact test for categorical variables, and the Student’s t-test for continuous variables. The variables that were identified to be correlated with CMV disease in the univariate logistic regression (P < 0.1) entered the multivariate logistic regression analysis. Cox proportional hazards regression models were used to analyze the risk factors of CMV disease. Kaplan-Meier survival curves were drawn to compare the all-cause mortality between patients with CMV disease and those without by using the log-rank test. The level of statistical significance was set to P < 0.05.

## Results

### Recipients’ characteristics

Three patients were excluded from the final analysis for missing the follow-up. A total of 308 patients were included in this study, including 302 patients from the Fist Affiliated Hospital of Guangzhou Medical University, 3 from the Fifth Affiliated Hospital of Sun Yat-Sen University, 2 from the People’s Hospital of Gaozhou, and 1 from the Shenzhen People’s Hospital (Table 1).

**Table 1:** Demographic and clinical characteristics of the patients (n = 308)

Variables	Number of patients	%
Male	260	84.4
Types of transplantation		
Double lung transplantation	108	35.1
Single lung transplantation	164	53.2
Heart–lung transplantation	17	5.5
Heart transplantation	19	6.2
Indications for transplantation		
Chronic obstructive pulmonary disease	102	34.4
Idiopathic interstitial lung disease	112	37.7
Connective tissue disease–related interstitial lung disease	17	5.7
Bronchiectasis	18	6.1
Occupational lung disease	19	6.4
Pulmonary hypertension	15	5.1
Dilated cardiomyopathy	10	2.9
Hypertrophic cardiomyopathy	4	1.2
Ischemic cardiomyopathy	3	0.9
Other	8	2.6
CMV serostatus		
D^+^/R^−^	19	6.2
D^+^/R^+^	233	75.7
D^−^/R^+^	36	11.7
D^−^/R^−^	20	6.5

*CMV* cytomegalovirus, *D* donor, *R* recipient

### CMV prophylaxis and time to CMV infection

A total of 288 patients received posttransplant CMV prophylaxis with intravenous ganciclovir for 2 to 3 weeks, except the 20 patients who died within 3 weeks posttransplant. However, only 98/288 (34.0%) patients adhered to oral valganciclovir for postdischarge CMV prophylaxis, with a median time of 60 days (range 15–180 days). Alternatively, in the other 190 (64.0%) patients who chose no postdischarge CMV prophylaxis, the plasma CMV viral loads were routinely monitored, and preemptive therapy was initiated once CMV DNAemia was diagnosed. The time from transplantation to the first detection of CMV DNAemia was significantly longer in the patients with postdischarge CMV prophylaxis than those without [median 98 days (range, 80–358 days) vs. median 55 days (range, 21–358 days), P < 0.01].

### CMV infection

A total of 1800 plasma samples and 600 BALF specimens were tested. Asymptomatic CMV DNAemia was found in 161 (52.3%) recipients. Tissue-invasive CMV disease was diagnosed in 50 (16.2%) patients, consisting of 42 patients with probable CMV pneumonia, 4 with proven CMV pneumonia, and 4 with CMV gastrointestinal disease. In addition, among these patients, there were 7 patients with probable CMV retinitis, and 2 with CMV myocarditis. CMV disease was managed with intravenous ganciclovir for 2 to 3 weeks, followed by oral valganciclovir [6].

Almost all the BALF specimens were from the lung and lung-heart recipients as bronchoscopy was not required for heart recipients if their clinical condition was stable. Among the 308 patients, CMV DNA was detected to be positive at least once in the BALF specimens in 203 (70.4%) patients.

### Risk factors of CMV disease

There were 8/19 (42%) cases of CMV disease in the D^+^/R^−^ patients, 38/233 (16%) cases in the D^+^/R^+^ patients, 3/36 cases (8%) in the D^−^/R^+^ patients, and 1/20 cases (5%) in the D^−^R^−^ group. Patients with CMV disease were significantly more likely to have a D^+^/R^−^ serostatus (P = 0.004) and r-ATG > 2 mg/kg (P = 0.002), but were significantly less likely to have valganciclovir prophylaxis (P = 0.02) compared to those without CMV disease (Table 2). Three independent risk factors of CMV disease were identified in the multivariate analysis, including the D^+^/R^−^ serostatus (odds ratio [OR]: 18.32; 95% confidence interval [CI]: 1.80-188.68, no valganciclovir prophylaxis (OR: 2.64; 95% CI: 1.05–6.64), and higher doses of rabbit anti-human thymocyte globulin (> 2 mg/kg) (OR: 4.25; 95% CI: 1.92–9.42) (Table 3).

**Table 2:** Comparison of the demographic and clinical characteristics between patients with and without CMV disease

Variables	Patients with CMV disease (n = 50)	Patients without CMV disease (n = 258)	P–value
Male, n (%)	29(58.0)	146 (56.6)	0.87
Age, year	53.4 ± 13.7	54.8 ± 14.4	0.94
Body mass index, kg/m^2^	20.0 ± 3.5	20.6 ± 4.0	0.74
Type of transplantation, n (%)			0.61
Bilateral lungs	18 (36)	90 (34.9)	
Single lung	28 (56)	136 (52.7)	
Heart–lung	3 (6)	14 (5.4)	
Heart	1 (2)	18 (7.0)	
Valganciclovir prophylaxis, n (%)	7 (14)	91 (35.3)	0.02
Duration of prophylaxis, day	39 (range, 12–193)	33 (range, 11–129)	0.04
D^+^/R^−^, n (%)	8 (16)	11 (4.26)	0.004
r–ATG > 2 mg/kg, n (%)	18 (36)	35 (13.6)	0.002
Basiliximab, n (%)	6 (12)	39 (15.1)	0.62
Plasma CMV viral load, UI/ml median (IQR)	31,500 (17,325–124,250)	2520 (1125–6325)	< 0.001

*CMV* cytomegalovirus, *D*^+^ CMV seropositive donors, *R*^−^ CMV seronegative recipients, *r-ATG* rabbit anti-human thymocyte globulin, *IQR* interquartile rage

**Table 3:** Risk factors of CMV disease

Variables	Univariate analysis		Multivariate analysis	
	OR (95% CI)	P value	OR (95% CI)	P value
Age	1.02 (1.00–1.05)	0.06	–	–
Sex (male vs. female)	1.80 (0.68–4.80)	0.24	–	–
Body mass index	1.57 (0.72–1.84)	0.57	–	–
Basiliximab (yes vs. no)	0.27 (0.06–1.15)	0.08	–	–
r–ATG (> 2 vs. ≤ 2 mg/kg)	3.39 (1.64–6.99)	0.001	4.25 (1.92–9.42)	0.001
Serostatus (D^+^/R^−^ vs. other)	13.82 (1.52–125.65)	0.02	18.32 (1.80–188.68)	0.014
No valganciclovir (true vs. false)	3.35 (1.45–7.74)	0.005	2.64 (1.05–6.64)	0.039

Heart-lung transplantation is included in double-lung transplantation. *CMV* cytomegalovirus, *D*^+^ CMV seropositive donors, *R*^−^ CMV seronegative recipients, *r-ATG* rabbit anti-human thymocyte globulin, *OR* odds ratio, *CI* confidence interval

### Survival and CMV disease outcomes

**Fig. 1:**
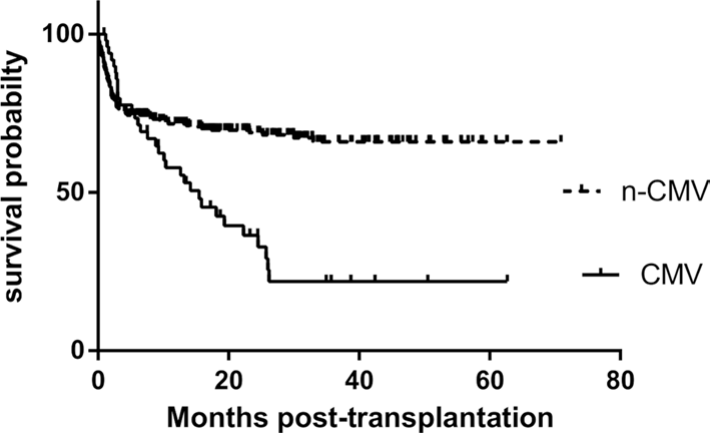
Kaplan-Meier survival curves comparing all-cause mortality between the patients with or without CMV disease (P = 0.03). *CMV* cytomegalovirus

The median follow-up time was 18.2 months (range, 2 to 70 months). The posttransplant 1-year all-cause mortality rate in the patients with CMV disease was significantly higher than those without it (42% vs. 22.5%, P = 0.03) (Fig. 1). Among the 50 patients who suffered from CMV disease, the causes of death were CMV pneumonia in 28 (56%) patients and opportunistic infection or other complications in 22 (44%) patients.

Four of the 5 suspected cases of ganciclovir-resistant CMV disease were confirmed by genotyping, with 3 cases only having *UL97* mutations, and 1 case having both *UL97* and *UL54* mutations. These patients were treated with high-dose ganciclovir or/and intravenous foscarnet. Of the 3 patients who had only *UL97* mutations, 2 patients died of severe respiratory failure, and 1 patient was successfully cured. The patient who had *UL54* mutations died of CMV gastrointestinal disease and post-transplant lymphoproliferative disorder.

## Discussion

Our study is the first investigation on the epidemiology of CMV infection in Chinese thoracic organ transplant recipients. The study highlights that both CMV viremia and CMV disease were prevalent among this cohort. Universal CMV prophylaxis benefitted the patients by reducing the CMV disease incidence. We also found that higher doses of r-ATG was a risk factor of CMV disease.

The overall CMV seropositive rate in our patients was 87.3%, which was consistent with previous data in liver and kidney transplant recipients in China [12, 13], and was similar to the worldwide CMV seroprevalence of 86% in solid organ donors [14, 15]. These patients had a moderate risk of posttransplant CMV infection. Only 6.2% (19/308) of our patients had the high-risk D^+^/R^−^ serostatus defined by the international guidelines [6], suggesting that only a few of the CMV seronegative recipients received grafts from seropositive donors in our cohort. In contrast, the overall CMV seroprevalence in the general population of the United States is 50% [16]. There is a large gap in CMV seroprevalence between countries and a differential CMV prophylaxis strategy may be more reasonable.

CMV DNA was detected in the BALF specimens in 70.4% (203/308) of our patients. However, 64.4% of the specimens were from the lung transplant recipients in whom CMV pneumonia was ruled out, indicating a low specificity of detecting BALF CMV DNA in diagnosing CMV pneumonia. According to the third international consensus guidelines on the management of CMV in solid-organ transplantation [6], measuring CMV DNA on BALF specimens is not a recommended practice and it didn’t contribute to the diagnosis of CMV pneumonia in our patients. Higher BALF CMV DNA levels are associated with an increased incidence of symptomatic CMV disease. Therefore, quantification of CMV DNA may potentially monitor subclinical viral replication [17–19]. However, this relationship was not investigated in our study because bronchoscopy was not required for patients who had no pulmonary symptoms or positive CT findings.

The D^+^/R^−^ patients constituted only 6.2% of our patients but contributed a disproportionate 16% to the patients with CMV disease. Unsurprisingly, both univariate and multivariate analyses showed that the D^+^/R^−^ serostatus was a risk factor of CMV disease. Consistent with previous studies [20–24], our finding further highlighted the D^+^/R^−^ serostatus as a primary risk factor of CMV infection in thoracic organ transplant recipients. Rapid viral DNA doubling is one of the contributing factors for the high CMV infection rate in the D^+^/R^−^ recipients [25]. Extended use of antiviral prophylaxis is recommended for CMV prevention in solid organ transplantation [6].

However, most of our patients did not adhere to the postdischarge valganciclovir therapy, with an adherence rate of 14% in the patients who developed CMV disease and an overall adherence rate of 31.8%. The duration of the postdischarge CMV prophylaxis was also relatively short. CMV prophylaxis can reduce the incidence of CMV disease [26, 27], which was also confirmed by our study. The low adherence rate of valganciclovir prophylaxis in Chinese patients may be associated with the high cost and the adverse effects of the drug.

Higher doses of r-ATG (≥ 2 mg/kg) were identified as a risk factor of CMV disease in our study. This finding was supported by previous studies showing the incidences of CMV reactivation had increased up to 10–50% since the introduction of r-ATG in solid organ transplantation [28–30]. However, our study didn’t find that basiliximab was associated with CMV disease, which was consistent with previous studies [31, 32].

Our study has limitations. Firstly, although CMV DNA was detected in the BALF specimens in a large proportion of our patients, the association between the BALF viral loads and the incidence of CMV pneumonia was not investigated due to the retrospective nature of our study. Secondly, the small number of high-risk D^+^/R^−^, lack of valganciclovir prophylaxis, and poor adherence to the therapy may compromise the representativeness of our study. Thirdly, the association between valganciclovir and patient survival was not analyzed. Fourthly, plasma CMV viral load was only monitored monthly after 6 months posttransplant, which may lead to delayed detection of DNAemia.

## Conclusion

The majority of the Chinese thoracic organ transplant recipients were at a moderate risk of CMV infection. However, the morbidity and mortality of CMV pneumonia in our patients were high, especially in those with the high-risk D^+^/R^−^ serostatus. Postdischarge CMV prophylaxis was effective in reducing the incidence of CMV disease. However, its use was significantly limited in our cohort. More medical resources are needed to address this issue in the Chinese patients.

## Data Availability

The datasets generated during the current study are available from the corresponding authors on reasonable request.

## Abbreviations

BALF: Bronchoalveolar lavage fluid
CI: Confidence interval
CMV: Cytomegalovirus
CT: Computed tomography
D^+^: Donor positive
DNA: Deoxyribonucleic acid
IgG: Immunoglobulin G
IQR: Inter quartile range
OR: Odds ratio
PCR: Polymerase chain reaction
R^−^: Recipient negative
r-ATG: Rabbit anti-human thymocyte globulin
